# Do diabetes and poor control of acute stress-related hyperglycemia increase the risk of ICU-acquired infections? A retrospective assessment in patients with septic shock

**DOI:** 10.1186/s13613-025-01596-z

**Published:** 2025-10-22

**Authors:** Alice Friol, Clément Devautour, Anna Semenov, Juliette Pelle, Marie Renaudier, Sarah Benghanem, Alain Cariou, Jean-Paul Mira, Julien Charpentier, Frédéric Pène

**Affiliations:** 1 Service de médecine intensive-réanimation, Hôpital Cochin, Assistance Publique - Hôpitaux de Paris, 27 rue du faubourg Saint-Jacques, Paris, 75014 France; 2 Université Paris Cité, Paris, France; 3 Institut Cochin, INSERM U1016, CNRS UMR 8104, Université Paris Cité, 22 rue Méchain, Paris, 75014 France

**Keywords:** Septic shock, Infection, Diabetes mellitus.

## Abstract

**Background:**

Patients with septic shock who survive the early resuscitation phase are prone to ICU-acquired infections. Although hyperglycemia harbors potent immunomodulatory properties, the impact of preexisting diabetes and the control of acute stress-induced hyperglycemia on the risk of further infections remains unclear.

**Materials and methods:**

We conducted a retrospective (2008–2023) single-center study in patients with septic shock who remained alive in the ICU after 72 h. Glycemic control was assessed during the first 72 h. Mild and severe hyperglycemia were defined by blood glucose levels > 8 mmol/L and > 10 mmol/L, respectively. Poor glycemic control was defined when blood glucose levels were above 8 mmol/L for more than 20% of time. The primary outcome was ICU-acquired infections.

**Results:**

The study involved 901 patients, with preexisting diabetes present in 22% of them. Most patients (71%) experienced hyperglycemic episodes > 8 mmol/L, prompting fast-acting insulin treatment. ICU-acquired infections developed in 243 patients (26.9%), with median time from ICU admission to diagnosis of 9 days, interquartile range [6–13]. There was no association between preexisting diabetes and ICU-acquired infections. Patients with further ICU-acquired infections displayed poorer control of stress-induced hyperglycemia, with longer exposure to hyperglycemia (78% with mild or severe hyperglycemia for more than 20% of time compared to 68% of patients without subsequent infections (*p* = 0.005)). Poor glycemic control was independently associated with the development of ICU-acquired infections.

**Conclusion:**

72-hour poor glycemic control, but not preexisting diabetes, was independently associated with an increased risk of ICU-acquired infections in septic shock patients and may therefore contribute to the post-aggressive immunosuppressive response. This argues for effective glycemic management to improve outcomes in this setting.

**Supplementary Information:**

The online version contains supplementary material available at 10.1186/s13613-025-01596-z.

## Introduction

Sepsis is a clinical syndrome characterized by a dysregulated systemic inflammatory response to an infection, leading to organ dysfunction. It is a common condition, affecting approximately 17 million people and accounting for a significant proportion of intensive care unit (ICU) admissions (30%) [1]. Septic shock, characterized by the combination of acute circulatory failure and tissue dysoxia, remains associated with dreadful crude mortality rates around 40% [2]. Thanks to timely recognition of sepsis, prompt initiation of antimicrobials and management of organ failure, most patients with septic shock now survive the initial resuscitation phase, but become exposed to ICU-acquired infections, which account as important causes of morbidity and mortality in this setting [3, 4].

Hyperglycemia is a common disorder in critically ill patients, related to preexisting diabetes and to the metabolic response to critical illness. Acute inflammatory disorders induce the release of endogenous corticosteroids and catecholamines and promote heightened gluconeogenesis and increased insulin resistance, resulting in stress-induced hyperglycemia in most patients, even though without preexisting diabetes. Chronic and acute hyperglycemia harbor various immunomodulatory properties towards both innate and adaptive immunity [5–8], raising the question of the contributions of preexisting diabetes mellitus and/or stress-induced hyperglycemia to the immunosuppressive response of sepsis. Studies that assessed the possible link between preexisting diabetes and ICU-acquired infections led to inconsistent results, either with an increased risk of bloodstream infections [9], or no significant association with of the occurrence of nosocomial infections [10]. Acute hyperglycemia warrants glycemic control through intensive insulin therapy in critically ill patients, though the optimal glycemic target remains questionable with respect to the hazard of iatrogenic hypoglycemia. Of note, the randomized trials that assessed intensive glycemic control in the ICU enrolled critically ill patients with various acute conditions, and did not focus on the primary outcome of ICU-acquired infections. So the specific impact of glycemic disturbances on the susceptibility to secondary infections has not been formally investigated in septic patients. To address this question, we carried out a retrospective study to address the impact of preexisting diabetes and/or the 72-hour glycemic control on the incidence of ICU-acquired infections in a cohort of patients with septic shock.

## Methods

### Patients and setting

We conducted a single-center retrospective observational study over a 16-year period (2008–2023) in a 24-bed medical ICU within a tertiary care facility. The study included all adult patients (18 years and older) with a primary diagnosis of septic shock within the first 48 h of admission. Patients who died or were discharged from the ICU within the initial 72 h were excluded to retain a population at risk of ICU-acquired infections. The study was conducted in accordance with the Helsinki declaration and was approved by the ethics committee of the French Intensive Care Society (Société de Réanimation de Langue Française, ref. CE SRLF 16–030) which waived the need for signed consent due to its retrospective design.

### Data collection

The following data were collected from the patient data management system (Clinisoft^®^, GE Healthcare): demographic information (age and gender), weight and size to calculate the body mass index, diabetes, immunocompromised status, primary source of infection. The severity at admission was assessed by various scoring systems including SOFA (Sequential Organ Failure Assessment) and SAPS2 (Simplified Acute Physiology Score 2) [11, 12], arterial lactate level, the requirement for invasive mechanical ventilation within the first 24 h. Daily records of glycemia, insulin dosages, and caloric intakes were collected over the first 72 h. This 72-h period encompasses the initial resuscitation phase in most patients and should avoid the concomitance with ICU-acquired infections that typically occur later on in this setting [4, 13]. The occurrence, the source and microbiological documentation of ICU-acquired infections were individually collected.

### Definitions

Sepsis, as defined by Sepsis-3, involved an acute increase in the SOFA score by 2 or more points, indicating organ dysfunction. Septic shock was characterized by persistent hypotension requiring vasopressor therapy to maintain a mean arterial pressure of 65 mmHg despite adequate fluid resuscitation, and increased arterial lactate level (> 2 mmol/L) [2]. This recent definition was also applied to patients managed before 2016. The diagnosis of preexisting diabetes relied on chronic antidiabetic therapy including oral medications and/or insulin. Chronic immunosuppression was defined by the presence of hematologic malignancy either active or in complete remission for less than 5 years, solid tumor under chemotherapy within the last 3 months or with metastasis, long-term immunosuppressive treatment, HIV infection at any stage, congenital immunosuppression, or corticosteroid therapy for a duration of more than 3 months at any dose or longer than 7 days at a dose ≥ 1 mg/kg prednisone-equivalent.

Acute stress-induced hyperglycemia was considered through the requirement of fast-acting insulin during the first 72 h following ICU admission. Based on previous studies and on our department’s protocol for management of glycemia [14, 15], we defined the following categories with respect to blood glucose levels: normal glycemia (3.3 to 8 mmol/L), whereas mild hyperglycemia (8 to 10 mmol/L), severe hyperglycemia (> 10 mmol/L), and hypoglycemia (< 3.3 mmol/L) [16]. Poor glycemic control was mainly defined as blood glucose levels above 8 mmol/L for more than 20% of time despite insulin therapy [17]. Subcutaneous or intravenous infusions of fast-acting insulin were administered to achieve a target glycemia below 8 mmol/L. All patients were managed according to a standardized insulin infusion protocol that remained similar throughout the 15-year study period. Blood glucose was measured primarily *via* capillary samples. Capillary glucose levels were measured every 3 h, and earlier in case of hypoglycemia. Insulin administration was adjusted by trained ICU nurses according to a written protocol (Table S1). Enteral nutrition was usually started within the first 48 h in the absence of contra-indications. With regard to potential changes in practices over the study period [18], the daily caloric intake was computed and served as an adjustment variable in multivariate analysis.

ICU-acquired infections were defined as suspected or microbiologically-confirmed infections occurring after at least 48 h of hospitalization in the ICU. To ensure diagnostic consistency over the 16-year inclusion period, ICU-acquired infections were retrospectively reviewed based on an uniform set of criteria aligned with international definitions. In particular hospital-acquired pneumonia, including ventilator-associated pneumonia were diagnosed according to the 2016 ATS/IDSA criteria and were characterized by new or progressive pulmonary infiltrates on chest radiographs, along with clinical and biological signs such as fever, purulent sputum, leukocytosis, and worsening oxygenation [19]. The microbiological documentation of ventilator-associated pneumonia mainly relied on semi-quantitative cultures of endotracheal aspirations. Catheter-related bloodstream infections were diagnosed by the growth of the same pathogen from both peripheral blood and catheter tip cultures, or from blood cultures drawn from the catheter and from venous puncture with a differential time to positivity > 120 min. Urinary tract infections were diagnosed upon the association of systemic manifestations of infection and positive urine bacterial culture at ≥ 10^5^ colony-forming-units per mL. Invasive fungal infections were diagnosed according to the current guidelines.

### Primary endpoint

The primary endpoint was the occurrence of a first episode of ICU-acquired infection.

### Statistical analysis

Determinants of ICU-acquired infection were assessed using both univariate and multivariate analyses. Continuous variables were expressed by median and interquartile ranges (IQR) and categorical variables in number (percentage). Differences between groups were assessed using Chi-square or Fisher’s exact test, the Mann-Whitney U test, and Student’s t-test as appropriate. The Fine and Gray method was used for multivariate analysis, completing a time-dependent cause-specific Cox proportional hazard model with discharge alive and death in ICU as competing events. The linearity of continuous covariates was formally assessed using restricted cubic splines with 4 knots at predefined 5th, 35th, 65th and 95th percentiles. Significant covariates in univariate analysis *(i.e.* with p-value inferior to 0.05) were assessed for multivariate analysis, as well as dynamic covariates occurring daily during hospitalization. Dynamic features were considered as discrete time-dependent covariates. Stepwise regression with Bayesian Information Criterion estimation was used to choose the model that provided the best fit to data. Collinearity was estimated by calculation of the generalized variance inflation factor of each covariate, and data with variance inflation factor superior to 2.5 were excluded from the model. All analysis were carried out with STATA (v.18) software. All tests were two-sided, with statistical significance defined as p-value of 0.05 or less.

## Results

### Cohort description

Over the 15-year study period, 1059 patients were admitted with the primary diagnosis of septic shock. After excluding those discharged early or deceased within 72 h, the core study cohort consisted of 901 patients who remained alive in the ICU after 72 h (Figure S1). The characteristics of the cohort are displayed in the Table 1. Preexisting diabetes mellitus was a frequent comorbid condition, present in 22% of patients. The main source of infection was the lung in 42% of patients. During the early 24 h, invasive mechanical ventilation was required in 83% and renal replacement therapy in 45%. The majority of patients (60%) received stress-dose hydrocortisone (200 mg per day) for vasopressor-dependent circulatory failure. In-ICU and in-hospital mortality rates were 30% and 40%, respectively (Table 2).

**Table 1:** Baseline and admission characteristics of patients with and without ICU-acquired infection

Variables	All patients	No ICU-acquired infection	ICU-acquired infection	*p*
Number of patients	901	658	243	
Demographics				
Age (years)	68 [57–77]	69 [57–78]	66 [56–76]	0.02
Male gender	550 (61)	389 (59)	161 (66)	0.05
Body mass index (kg/m^2^)	24.3 [21.5–27.7]	24.3 [21.6–27.7]	24.3 [21.5–27.6]	0.94
Obesity (body mass index > 30 kg/m^2^)	98 (11)	71 (11)	27 (11)	0.89
Comorbidities				
Diabetes mellitus	200 (22)	146 (22)	54 (22)	0.99
Chronic heart failure	160 (18)	123 (19)	37 (15)	0.23
Chronic Respiratory Disease	119 (13)	88 (13)	31 (13)	0.81
Chronic renal failure	119 (13)	99 (15)	20 (8)	0.007
Immunodeficiency	116 (13)	267 (41)	94 (39)	0.61
Cirrhosis	101 (11)	70 (11)	31 (13)	0.37
Characteristics of sepsis at admission				
Hospital-acquired	120 (13)	248 (38)	104 (43)	0.16
Source of infection				0.0001
Lung	381 (42)	246 (37)	135 (56)	
Urinary tract	132 (15)	118 (18)	14 (6)	
Gastrointestinal tract	184 (20)	130 (20)	54 (22)	
Primary bacteremia	53 (6)	42 (6)	11 (5)	
Central nervous system	16 (2)	12 (2)	4 (2)	
Skin and soft tissue	48 (5)	38 (6)	10 (4)	
Unknown	57 (6)	50 (8)	7 (3)	
Microbiological documentation	651 (72)	476 (72)	175 (72)	0.92
Positive blood culture	310 (34)	227 (35)	83 (34)	0.92
Severity at admission				
SOFA score (points)	9 [6–13]	9 [6–12]	11 [7–13]	0.002
SAPS 2 (points)	70 [54–85]	68 [53–83]	76 [60–89]	0.0001
Arterial lactate level (mmol/L)	4.1 [3-6.8]	4.1 [2.9–6.6]	4.5 [3-7.8)]	0.03
Stress-dose hydrocortisone	542 (60)	379 (58)	163 (67)	0.01

**Table 2:** ICU management and outcomes of patients with and without ICU-acquired infection

Variables	All patients	No ICU-acquired infection	ICU-acquired infection	*p*
Number of patients	901	658	243	
ICU management				
Invasive mechanical ventilation	745 (83)	507 (77)	238 (98)	0.0001
Renal replacement therapy	407 (45)	254 (39)	153 (63)	0.0001
72-hour cumulated dose of norepinephrine (mg)	69 [26–177]	60 [23–146]	117 [47–242]	0.0001
72-hour glycemic control				
Blood glucose level (mmol/L)	7.4 [6.4–8.6]	7.4 [6.4–8.6]	7.5 [6.6–8.7]	0.11
Quartiles of median blood glucose levels				0.24
Q1: 4.2–6.4 mmol/L	226 (25)	175 (27)	49 (20)	
Q2: 6.4–7.4 mmol/L	225 (25)	159 (24)	67 (28)	
Q3: 7.4–8.6 mmol/L	225 (25)	163 (25)	62 (26)	
Q4: 8.6–14.8 mmol/L	226 (25)	161 (24)	65 (27)	
Time with mild or severe hyperglycemia (h)	37 [16–61]	36 [14–60]	41 [23–64]	0.03
Mild or severe hyperglycemia for > 20% of time	637 (71)	448 (68)	189 (78)	0.005
Time with severe hyperglycemia (h)	11 [0–28]	11 [0–27]	14 [1–31]	0.01
Severe hyperglycemia for > 20% of time	313 (35)	219 (33)	94 (39)	0.12
Caloric intake (kcal/kg/day)	3.9 [1.6-7.0]	3.6 [1.4–6.7]	4.7 [2.1–7.4]	0.001
Fast-acting insulin dosage (UI/kg/h)	0.003 [0-0.014]	0.002 [0-0.01]	0.004 [0-0.02]	0.02
Weight-adjusted insulin D1-D3 > 0.01 UI/kg/h	292 (32)	203 (31)	89 (37)	0.10
Hypoglycemia	187 (21)	129 (20)	58 (24)	0.16
Outcomes				
Duration of invasive mechanical ventilation, days	7 [3–12]	5 [3–8]	16 [9–25]	0.0001
Length of ICU stay, days	8 [5–16]	7 [4–10]	21 [13–32]	0.0001
Therapeutic limitations	231 (26)	146 (22)	85 (35)	0.0001
In-ICU mortality	270 (30)	167 (25)	103 (42)	0.0001
In-hospital mortality	337 (40)	214 (35)	123 (53)	0.0001

Mild and severe hyperglycemia denote blood glucose levels > 8 mmol/L and > 10 mmol/L, respectively

### 72-hour glycemic control

The median blood glucose level during the first 72 h was 7.4 mmol/L [6.4–8.6]. 71% of patients experienced poor glycemic control (i.e. blood glucose levels > 8 mmol/L for > 20% of time). Median times spent above 8 mmol/L and above 10 mmol/L were 37 h [IQR 16–61] and 11 h [IQR 0–28], respectively. 21% of patients experienced at least one hypoglycemic episode (Table 2).

### Features of ICU-acquired infections

ICU-acquired infections occurred in 243 patients (26.9%), diagnosed after a median of 9 days [6–13] of ICU stay (Table 3). 95% of ICU-acquired infections occurred after three days. Patients with ICU-acquired infections were significantly younger (66 [56–76] vs. 69 [57–78] years, *p* = 0.02), displayed higher severity at admission with higher requirements of hydrocortisone treatment, invasive mechanical ventilation and renal replacement therapy. ICU-acquired pneumonia occurred mainly in patients with primary pulmonary infections (75% of episodes). Of note, no differences were observed with regard to prior comorbid conditions including diabetes, obesity, or immunosuppression (Table 1). The occurrence of ICU-acquired infections was associated with worsened outcomes, including prolonged mechanical ventilation (16 [9–25] vs. 5 [3–8] days, *p* = 0.0001) and ICU stay (21 [13–32] vs. 7 [4–10] days, *p* = 0.0001), and higher in-ICU and in-hospital mortality rates (Table 2).

**Table 3:** Characteristics of the first ICU-acquired infection

	First ICU-acquired infection
Number of patients	243
**Source of infection**	
Lung	109 (45)
Gastrointestinal tract	38 (16)
Primary bacteremia	58 (24)
Skin and soft tissue	12 (5)
Osteo-articular	6 (2)
Urinary tract	4 (2)
Unknown	13 (5)
Microbiological documentation	242 (99.5)
Positive blood culture	77 (32)
**Clinical severity on the day of infection**	
SOFA score (points)	10 [7–13]
Vasopressor support	141 (58)
Lactate level (mmol/L)	1.6 [1.1–2.3]
Invasive mechanical ventilation	142 (65)
Renal replacement therapy	42 (18)

### Hyperglycemia and ICU-acquired infections

**Fig. 1:**
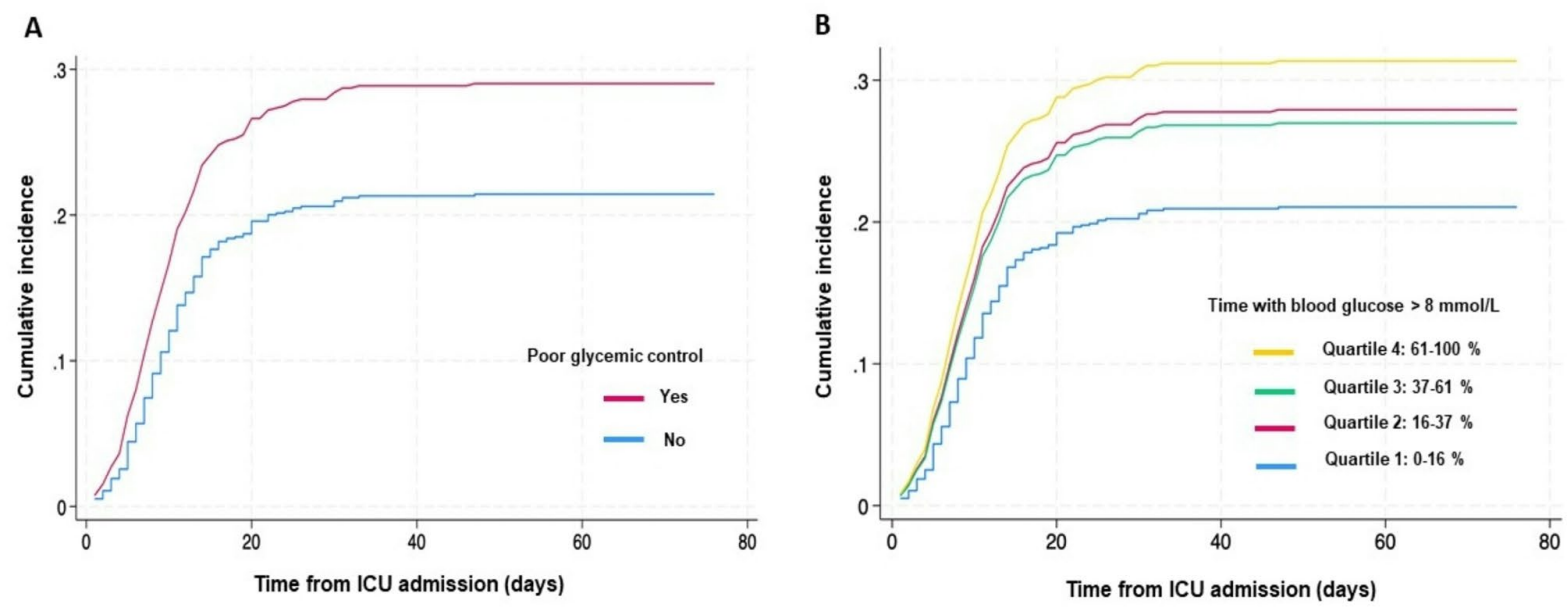
Cumulative incidences of ICU-acquired infections according to 72-hour glycemic control. Poor glycemic control was defined by blood glucose level > 8 mmol/L for more than 20% of time (A) and the proportions of time spent above 8 mmol/L separated into quartiles (B). Curves were built through a multivariate competing-risk analysis taking into account the competitive risks of death and ICU discharge and adjusted on the following variables: age, preexisting diabetes, admission SOFA score, pneumonia on admission, intubation on a given day, presence of intravascular central venous, arterial or dialysis catheters on a given day, transfusion of blood products on a given day, daily caloric intake

Patients with further ICU-acquired infection exhibited prolonged periods with mild and severe hyperglycemia within the first 72 h. Among patients with ICU-acquired infections, 78% spent more than 20% of time with mild or severe hyperglycemia as compared to 68% for patients who remained free of further infection (*p* = 0.005). Time spent with mild or severe hyperglycemia was associated with increased cumulated incidences of ICU-acquired infection (Fig. 1). Higher insulin doses were required in patients with ICU-acquired infection (*p* = 0.02). No difference in hypoglycemia was observed between the groups (Table 2).

The association between 72-hour glycemic control and ICU-acquired infections was assessed using multivariate cause-specific hazard models adjusted for age, admission SOFA score, pneumonia on admission, intubation on a given day, presence of intravascular central venous, arterial or dialysis catheters on a given day, transfusion of blood products on a given day, daily caloric intake. The presence of preexisting diabetes was forced into the model, yet withoutassociation with the development of ICU-acquired infections. Several metrics related to 72-hour glycemic control were investigated, including blood glucose level > 8 mmol/L for > 20% of time, but also median blood glucose level (as a continuous and categorical variable) and percentage of time spent > 8 mmol/L or > 10 mmol/L (table S2). Higher median blood glucose level and prolonged time spent above 8 mmol/L remained independently associated with ICU-acquired infections, with the strongest association observed in the highest quartile of hyperglycemia exposure (Table 4; Fig. 1). There were no significant interactions between pre-existing diabetes and glucose control metrics (table S2). In addition, we conducted subgroup analysis with regard to preexisting diabetes (Table S3). Poor glycemic control remained significantly associated with ICU-acquired infections in non-diabetic patients, whereas not significant in diabetic patients (Figure S2). In a sensitivity analysis with a landmark set at day 4 (following the 72-hour glycemic control intervention), poor glycemic control remained independently associated with further occurrence of ICU-acquired infections (table S4).

**Table 4:** Associations between 72-hour glycemic control and the development of ICU-acquired infections (multivariate analysis)

	aCSH	95% confidence interval		*p*
72-h median blood glucose level (+ 1 mmol/L)				
	1.09	1.01	1.18	0.021
72-h median blood glucose level (quartiles)				
Q1: < 6.4 mmol/L	Ref.	Ref.	Ref.	Ref.
Q2: 6.4–7.4 mmol/L	1.59	1.09	2.32	0.017
Q3: 7.4–8.6 mmol/L	1.34	0.91	1.95	0.136
Q4: >8.6 mmol/L	1.53	1.03	2.27	0.034
72-h blood glucose level > 8 mmol/L for > 20% of time				
	1.42	1.03	1.97	0.034
Time duration with blood glucose levels > 8 mmol/L over 72 h (quartiles)				
Q1: 0–16%	Ref.	Ref.	Ref.	Ref.
Q2: 16–37%	1.38	0.94	2.05	0.104
Q3: 37–61%	1.33	0.90	1.97	0.155
Q4: 61–100%	1.59	1.07	2.37	0.022

Multivariate competing-risk analysis adjusted on the following variables: age, preexisting diabetes, admission SOFA score, pneumonia on admission, intubation on a given day, presence of intravascular central venous, arterial or dialysis catheters on a given day, transfusion of blood products on a given day, daily caloric intake. There were no missing data

Likelihood ratio tests of the non-linear components were non-significant (*p* > 0.05) for all continuous variables, supporting the linear hypothesis

aCSH: adjusted cause-specific hazard

## Discussion

The significance of hyperglycemia on infection control in septic shock is incompletely understood. We herein report that 72-hour poor glycemic control of acute stress-induced hyperglycemia, but not preexisting diabetes, was independently associated with the further development of ICU-acquired infections. The interpretation of these findings is questionable, since the ability to achieve the control of acute hyperglycemia may be merely viewed as a marker of severity but may also further contribute to the immunosuppressive response to sepsis.

Both chronic and acute hyperglycemia are likely to impact on immune functions. In diabetic patients, chronic hyperglycemia has been shown to impair innate and adaptive immune responses, associated with increased susceptibility towards infections. Such hyperglycemia-related immune dysfunctions include T-cell defects through decreased expression of class I major histocompatibility complex and decreased production of interferon-γ, under the influence of advanced glycation end products [20], reduced HLA-DR expression on monocytes [5, 21], and alterations in chemotaxis and phagocytosis functions by neutrophils and monocytes [22–24]. Acute stress hyperglycemia resulting from the transient metabolic response to aggression can promote oxidative stress, impair neutrophil functions, disrupt complement activation [25], and alter lymphocyte homeostasis. A few studies in critically ill patients have identified correlations between acute hyperglycemia and biomarkers of post-aggressive immunosuppression, including glucose-to-lymphocytes ratio [6], reduced HLA-DR expression on monocytes [8], and dysregulated cytokine release [7]. Furthermore, insulin itself may be immunomodulatory by anti-inflammatory effects or by promoting anti-infective host responses during sepsis [25].

The immune pathophysiology of sepsis is characterized by concurrent pro-inflammatory and immunosuppressive responses [26, 27], often resulting in sustained immunoparalysis, where acquired functional defects in innate and adaptive immune cells have been associated with increased susceptibility to ICU-acquired infections [28–31]. Beyond the severity of the primary insult, most therapeutic interventions in the ICU, including invasive devices, transfusions, corticosteroids, sedatives, catecholamines, invasive mechanical post ventilation and extra-corporeal circulations, are also likely to impair local and systemic defense mechanisms. Along this line, the present results suggest that acute hyperglycemia may also impair the further anti-infective responses in patients resuscitated from septic shock. A link between glucose metabolism and post-septic immunosuppression was suggested by the reduced expression of genes associated with glycolysis in circulating mononuclear cells from septic patients with ICU-acquired infections [4]. Altogether, the compilation of clinical and biological observations suggests that dysregulated immunometabolism may drive immune defects and thereby contribute to the immunoparalysis observed in critically ill patients.

Current guidelines, as per the 2021 Surviving Sepsis Campaign recommendations, suggest initiating insulin therapy for adults with sepsis or septic shock when glucose levels reach or exceed 180 mg/dL (10 mmol/L) [32]. This guideline is supported by strong, moderate-quality evidence and was developed from several randomized controlled trials that addressed glycemic control protocols in the ICU, primarily focusing on mortality outcomes [33–35] These studies compared intensive insulin therapy, aiming for blood glucose levels between 80 and 110 mg/dL (4.4–6.1 mmol/L) [33, 35], with conventional insulin therapy initiated when blood glucose levels exceeded 140–180 mg/dL (7.8–10 mmol/L) [33, 36] or 180–215 mg/dL (10.0-11.9 mmol/L) [33, 35]. The prevalence of preexisting diabetes was generally around 20%. Sepsis accounted for the acute condition in variable proportions of patients ranging from 20% to 50%. Overall, glucose control by intensive insulin therapy did not result in survival improvement. As secondary or tertiary endpoint, the incidence of secondary infections, collected as any new infection [35] or restricted to ICU-acquired bacteremia [33, 34], or indirectly estimated from the number of febrile days [36] or exposure to antibiotics [33, 36], was not different between conventional and intensive glucose control. Altogether, those studies did not evidence any impact of strict glycemic control on the incidence of ICU-acquired infections, but the heterogeneity of populations and the various definitions of infection applied preclude any definite conclusions.

The main limitation of this study lies in its retrospective design, which hinders the accuracy of diagnosis of ICU-acquired infections the causality inference. The multivariate analysis allowed us to conclude to an independent association between acute hyperglycemia metrics and the risk of further ICU-acquired infections, but cannot establish a definite causal relationship. However, the adjustment for multiple common confounders thanks to the size of the cohort, and the time interval between the intervention (72-h glycemic control) and the endpoint (ICU-acquired infection) both argue for a plausible causal relationship with low risk of reverse causality. It was a single-center study, which raises concerns about its external validity. The relatively high incidence (26.9%) of ICU-acquired infections should be interpreted in light of a selected cohort at risk of 72-hour survivors resuscitated from septic shock. Our analysis focused on markers of acute hyperglycemia, but alternative markers like the stress hyperglycemia ratio that combines the admission glucose levels and the HbA1c value might be of interest by taking into account both chronic and acute hyperglycemia [37]. However, the high number of patients without HbA1c measurements precluded any meaningful analysis of this parameter. The impact of persistent stress-related hyperglycemia beyond 72 h was not assessed since often concurrent to superimposed ICU-acquired complications, hence with a potential hazard of reverse causality. Glucose levels were generally measured from capillary puncture using point-of-care devices, which minimizes blood loss, but may not be fully accurate in critically ill patients when compared to reference measurements from arterial or venous samples in the central laboratory [38]. Finally, although we assume that poor glycemic control may contribute to the post-aggressive immunosuppressive response, immune biomarkers were not available to establish a biological link between hyperglycemia and ICU-acquired immune dysfunction.

## Conclusion

Our study highlights a prominent role of the 72-hour control of stress-induced hyperglycemia, associated with the risk of ICU-acquired infections in septic shock patients, which may therefore contribute to the immunosuppressive response of sepsis. This suggests that effective glycemic management may be an actionable intervention to reduce the incidence of ICU-acquired infections in critically ill patients.

## Supplementary Information

Supplementary Material 1.

## Data Availability

The datasets generated and/or analyzed during the current study are not publicly available due to French regulations but are available from the corresponding author on reasonable request. Declarations.

## Abbreviations

CSH: Cause-specific hazard
HIV: Human Immunodeficiency Virus
ICU: Intensive care unit
IQR: Interquartile Range
SAPS2: Simplified Acute Physiology Score 2
SOFA: Sequential Organ Failure Assessment
