# Supplementary material for: Do diabetes and poor control of acute stress-related hyperglycemia increase the risk of ICU-acquired infections? A retrospective assessment in patients with septic shock
Source: Ann Intensive Care. 2025 Oct 22;15:168. doi: 10.1186/s13613-025-01596-z (PMC12546208; doi:10.1186/s13613-025-01596-z)
Supplement: Supplementary file 1 — Supplementary Material 1. [file 13613_2025_1596_MOESM1_ESM.docx]

**Do diabetes and acute stress-related hyperglycemia increase the risk of ICU-acquired infections? A retrospective assessment in patients with septic shock**

Alice Friol, Clément Devautour, Anna Semenov, Juliette Pelle, Marie Renaudier,

Sarah Benghanem, Alain Cariou, Jean-Paul Mira, Julien Charpentier, Frédéric Pène

**Supplementary materials**

**Table S1**: Protocol for insulin therapy in the ICU

**Table S2**: Multivariate competing-risk cause-specific hazard models for risk factors associated with ICU-acquired infections.

**Table S3**: Multivariate competing-risk cause-specific hazard model for risk factors associated with ICU-acquired infections with regard to preexisting diabetes.

**Table S4**: Multivariate competing-risk cause-specific hazard models for risk factors associated with ICU-acquired infections – Landmark sensitive analysis starting after the initial 72-hour period of glycemic control

**Figure S1**: Flowchart of the study

**Figure S2**: Cumulative incidences of ICU-acquired infections in non-diabetic (n=701) and diabetic patients (n=200) according to 72-hour glycemic control.

| **Subcutaneous fast-acting insulin administration protocol**  *Based on 6-hour glucose measurements.* | | | |
| --- | --- | --- | --- |
| **Glycemia** | |  | |
| < 8 mmol/L | | No insulin | |
| 8 – 10 mmol/L | | 4 IU | |
| 10 – 15 mmol/L | | 6 IU | |
| 1. – 20 mmol/L | | 8 IU | |
| > 20 mmol/L | | 10 IU | |
| **Continuous intravenous fast-acting insulin infusion: initiation protocol (when blood glucose sustains ≥ 10 mmol/L)**  *Monitor blood glucose after 1 hour and adjust accordingly.* | | | |
| **Glycemia** | **First i.v. injection** | | **Continuous i.v. infusion** |
| 10 - 12 mmol/L | 2 IU | | 2 IU/h |
| 12 - 14 mmol/L | 3 IU | | 3 IU/h |
| 14 - 16 mmol/L | 4 IU | | 4 IU/h |
| **Continuous intravenous fast-acting insulin infusion: maintenance protocol**  *Blood glucose monitoring 1 hour after any change in infusion rate, and every 3 hours otherwise.* | | | |
| **Glycemia** | **Infusion rate adjustment** | | **Maximum dosage rate** |
| 5 - 6 mmol/L | 0 IU/h | | 1 IU/h |
| 6 - 7 mmol/L | 0 IU/h | | 1.5 IU/h |
| 7 - 8 mmol/L | 0 IU/h | | 2 IU/h |
| 8 - 10 mmol/L | 1 IU/h | | 3 IU/h |
| 10 - 12 mmol/L | 1.5 IU/h | | 4 IU/h |
| 12 - 15 mmol/L | 2 IU/h | | 6 IU/h |
| > 15 mmol/L | 2.5 IU/h | | 8 IU/h |

**Table S1**: Protocol for insulin therapy in the ICU

*IU: international units*

**Table S2**: Multivariate competing-risk cause-specific hazard models for risk factors associated with ICU-acquired infections.

|  | **aCSH** | **95% confidence interval** | | ***p*** |  |
| --- | --- | --- | --- | --- | --- |
| **Model 1** |  | | | |  |
|  |  |  |  |  |  |
| Age (+ 1 year) | 0.99 | 0.99 | 1.00 | 0.148 |  |
| Admission SOFA (+1 point) | 1.03 | 1.00 | 1.06 | 0.085 |  |
| Preexisting diabetes | 0.98 | 0.71 | 1.36 | 0.918 |  |
| Pneumonia on admission | 1.63 | 1.26 | 2.11 | <0.001 |  |
| Hydrocortisone therapy | 1.22 | 0.93 | 1.59 | 0.15 |  |
| Intubation one given day | 2.45 | 1.38 | 4.35 | 0.002 |  |
| Arterial catheter one given day | 1.22 | 0.65 | 2.31 | 0.538 |  |
| Central venous catheter one given day | 1.10 | 0.47 | 2.59 | 0.831 |  |
| Dialysis catheter one given day | 1.03 | 0.77 | 1.38 | 0.848 |  |
| Transfusion of blood products one given day | 1.94 | 1.40 | 2.68 | <0.001 |  |
| Caloric intake (+1 kcal/kg/day) one given day | 1.02 | 1.01 | 1.03 | 0 |  |
| Median blood glucose level (+1 mmol/L) | 1.09 | 1.01 | 1.18 | 0.021 |  |
| **Model 2** |  | | | |  |
|  |  |  |  |  |  |
| Age (+ 1 year) | 0.99 | 0.99 | 1.00 | 0.203 |  |
| Admission SOFA (+1 point) | 1.02 | 1.00 | 1.05 | 0.095 |  |
| Preexisting diabetes | 1.03 | 0.76 | 1.41 | 0.834 |  |
| Pneumonia on admission | 1.63 | 1.26 | 2.11 | <0.001 |  |
| Hydrocortisone therapy | 1.22 | 0.93 | 1.59 | 0.15 |  |
| Intubation one given day | 2.51 | 1.40 | 4.48 | 0.002 |  |
| Arterial catheter one given day | 1.21 | 0.64 | 2.29 | 0.548 |  |
| Central venous catheter one given day | 1.11 | 0.47 | 2.59 | 0.814 |  |
| Dialysis catheter one given day | 1.02 | 0.76 | 1.37 | 0.876 |  |
| Transfusion of blood products one given day | 1.94 | 1.40 | 2.68 | <0.001 |  |
| Caloric intake (+1 kcal/kg/day) one given day | 1.02 | 1.01 | 1.03 | <0.001 |  |
| Median blood glucose level |  |  |  |  |  |
| Quartile 1 : < 6.4 mmol/L | Ref. | Ref. | Ref. |  |  |
| Quartile 2 : 6.4-7.4 mmol/L | 1.59 | 1.09 | 2.32 | 0.017 |  |
| Quartile 3 : 7.4-8.6 mmol/L | 1.34 | 0.91 | 1.95 | 0.136 |  |
| Quartile 4 : > 8.6 mmol/L | 1.53 | 1.03 | 2.27 | 0.034 |  |

*aCSH: adjusted cause-specific hazard.*

*p-values for interaction between preexisting diabetes and glucose levels:*

*model 1: 0.75*

*model 2: 0.65, 0.91 and 0.69*

|  | **aCSH** | **95% confidence interval** | | ***p*** |
| --- | --- | --- | --- | --- |
| **Model 3** | | | | |
| Age (+ 1 year) | 0.99 | 0.99 | 1.00 | 0.127 |
| Admission SOFA (+1 point) | 1.02 | 1.00 | 1.05 | 0.098 |
| Preexisting diabetes | 1.01 | 0.74 | 1.38 | 0.946 |
| Pneumonia on admission | 1.62 | 1.25 | 2.10 | <0.001 |
| Hydrocortisone therapy | 1.20 | 0.92 | 1.57 | 0.177 |
| Intubation one given day | 2.39 | 1.34 | 4.26 | 0.003 |
| Arterial catheter one given day | 1.25 | 0.66 | 2.36 | 0.487 |
| Central venous catheter one given day | 1.11 | 0.47 | 2.60 | 0.815 |
| Dialysis catheter one given day | 1.01 | 0.75 | 1.36 | 0.93 |
| Transfusion of blood products one given day | 1.94 | 1.40 | 2.68 | <0.001 |
| Caloric intake (+1 kcal/kg/day) one given day | 1.02 | 1.01 | 1.03 | <0.001 |
| Blood glucose level > 8 mmol/L for > 20% of time | 1.42 | 1.03 | 1.97 | 0.034 |
| **Model 4** |  | | | |
| Age (+ 1 year) | 0.99 | 0.99 | 1.00 | 0.134 |
| Admission SOFA (+1 point) | 1.02 | 0.99 | 1.05 | 0.11 |
| Preexisting diabetes | 0.99 | 0.72 | 1.36 | 0.947 |
| Pneumonia on admission | 1.63 | 1.26 | 2.11 | <0.001 |
| Hydrocortisone therapy | 1.19 | 0.91 | 1.56 | 0.205 |
| Intubation one given day | 2.44 | 1.38 | 4.34 | 0.002 |
| Arterial catheter one given day | 1.24 | 0.66 | 2.35 | 0.499 |
| Central venous catheter one given day | 1.08 | 0.46 | 2.55 | 0.856 |
| Dialysis catheter one given day | 1.02 | 0.76 | 1.36 | 0.919 |
| Transfusion of blood products one given day | 1.95 | 1.41 | 2.69 | <0.001 |
| Caloric intake (+1 kcal/kg/day) one given day | 1.02 | 1.01 | 1.03 | <0.001 |
| Time duration with blood glucose levels > 8 mmol/L |  |  |  |  |
| Quartile 1 : 0-16 % | Ref. | Ref. | Ref. |  |
| Quartile 2 : 16-37 % | 1.38 | 0.94 | 2.05 | 0.104 |
| Quartile 3 : 37-61 % | 1.33 | 0.90 | 1.97 | 0.155 |
| Quartile 4 : 61-100 % | 1.59 | 1.07 | 2.37 | 0.022 |

**Table S2** (continued): Multivariate competing-risk cause-specific hazard models for risk factors associated with ICU-acquired infections.

*aCSH: adjusted cause-specific hazard.*

*p-values for interaction between preexisting diabetes and glucose levels:*

*model 3: 0.31*

*model 4: 0.17, 0.33 and 0.46*

|  | **aCSH** | **95% confidence interval** | | ***p*** |
| --- | --- | --- | --- | --- |
| Age (+ 1 year) | 0.99 | 0.99 | 1.002 | 0.112 |
| Admission SOFA (+1 point) | 1.02 | 0.995 | 1.05 | 0.112 |
| Pneumonia on admission | 1.61 | 1.24 | 2.09 | 0.000 |
| Hydrocortisone therapy | 1.19 | 0.91 | 1.56 | 0.192 |
| Intubation one given day | 2.38 | 1.34 | 4.25 | 0.003 |
| Arterial catheter one given day | 1.25 | 0.66 | 2.36 | 0.488 |
| Central venous catheter one given day | 1.11 | 0.47 | 2.60 | 0.810 |
| Dialysis catheter one given day | 1.01 | 0.75 | 1.35 | 0.972 |
| Transfusion of blood products one given day | 1.93 | 1.40 | 2.67 | <0.001 |
| Caloric intake (+1 kcal/kg/day) one given day | 1.02 | 1.01 | 1.03 | 0.001 |
| Blood glucose level > 8 mmol/L for > 20% of time |  |  |  |  |
| in non-diabetic patients | 1.51 | 1.07 | 2.13 | 0.018 |
| in diabetic patients | 0.61 | 0.24 | 1.58 | 0.310 |

**Table S3**: Multivariate competing-risk cause-specific hazard model for risk factors associated with ICU-acquired infections with regard to preexisting diabetes.

*aCSH: adjusted cause-specific hazard*

**Table S4**: Multivariate competing-risk cause-specific hazard models for risk factors associated with ICU-acquired infections – Landmark sensitive analysis starting after the initial 72-hour period of glycemic control

|  | **aCSH** | **95% confidence interval** | | ***p*** |
| --- | --- | --- | --- | --- |
| **Model 3** | | | | |
| Age (+ 1 year) | 0.99 | 0.98 | 1.00 | 0.124 |
| Admission SOFA (+1 point) | 1.01 | 0.99 | 1.05 | 0.328 |
| Preexisting diabetes | 2.30 | 0.80 | 6.59 | 0.120 |
| Pneumonia on admission | 1.65 | 1.25 | 2.17 | <0.001 |
| Hydrocortisone therapy | 1.21 | 0.91 | 1.60 | 0.187 |
| Intubation one given day | 2.41 | 1.33 | 4.38 | 0.004 |
| Arterial catheter one given day | 1.37 | 0.70 | 2.67 | 0.359 |
| Central venous catheter one given day | 0.96 | 0.40 | 2.32 | 0.929 |
| Dialysis catheter one given day | 1.12 | 0.83 | 1.52 | 0.461 |
| Transfusion of blood products one given day | 2.03 | 1.44 | 2.86 | <0.001 |
| Caloric intake (+1 kcal/kg/day) one given day | 1.02 | 1.01 | 1.03 | <0.001 |
| Blood glucose level > 8 mmol/L for > 20% of time | 1.51 | 1.05 | 2.18 | 0.026 |

*aCSH: adjusted cause-specific hazard.*

**
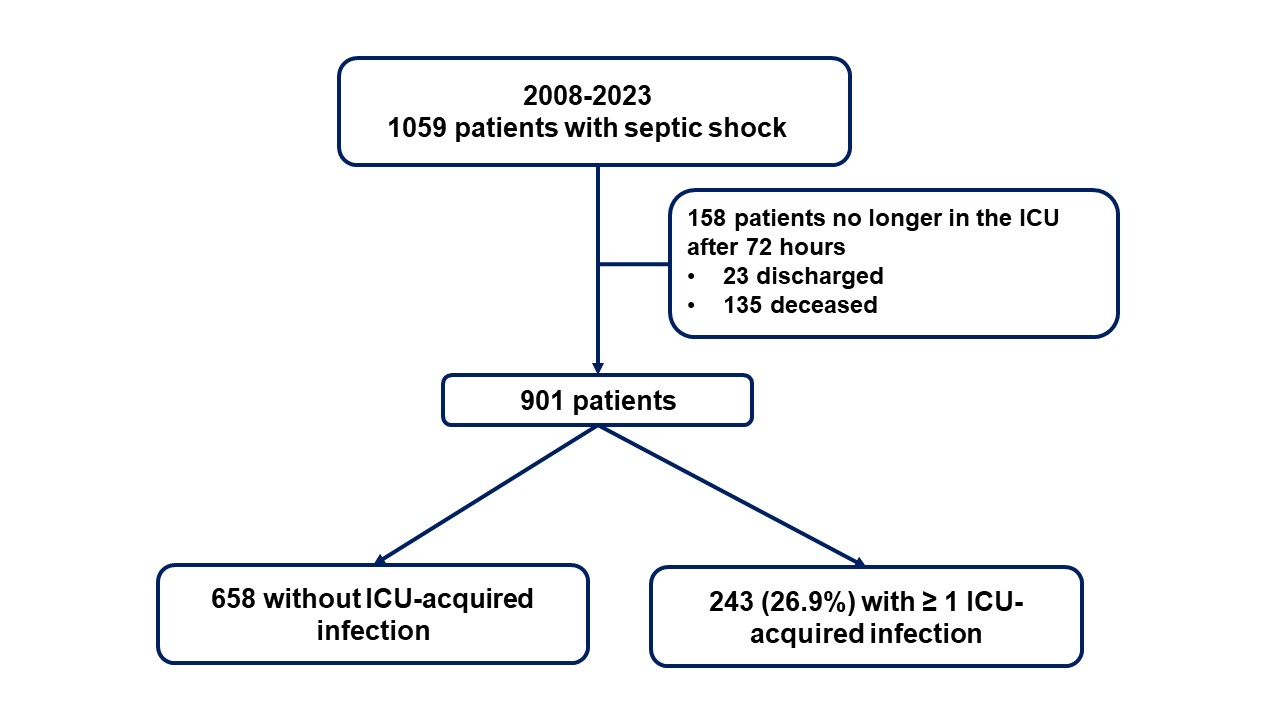
**

**Figure S1**: Flowchart of the study

**
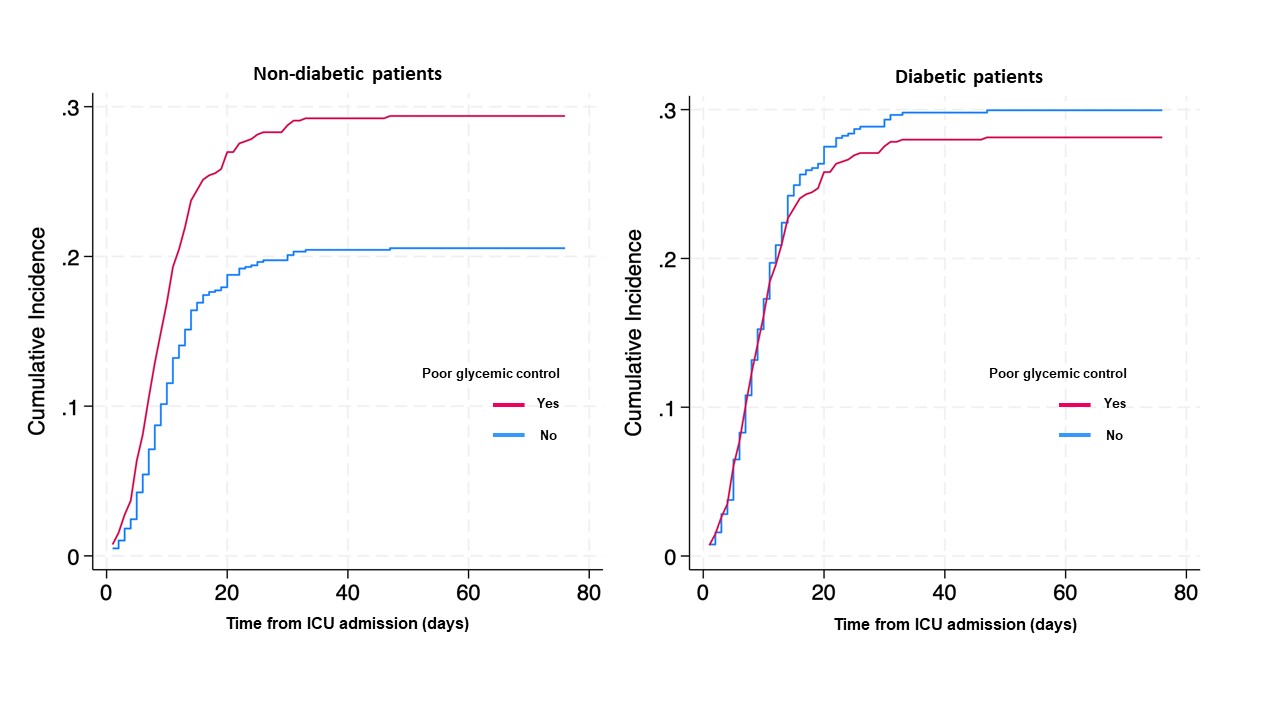
**

**Figure S2: Cumulative incidences of ICU-acquired infections in non-diabetic (n=701) and diabetic (n=200) patients according to 72-hour glycemic control.**

*Poor glycemic control was defined by blood glucose level > 8 mmol/L for more than 20% of time. Curves were built through a multivariate competing-risk analysis taking into account the competitive risks of death and ICU discharge and adjusted on the following variables: age. admission SOFA score. pneumonia on admission, intubation on a given day, presence of intravascular central venous, arterial or dialysis catheters on a given day, transfusion of blood products on a given day, daily caloric intake.*
